# Targeting Gut–Lung Crosstalk in Acute Respiratory Distress Syndrome: Exploring the Therapeutic Potential of Fecal Microbiota Transplantation

**DOI:** 10.3390/pathogens14121206

**Published:** 2025-11-26

**Authors:** Mairi Ziaka

**Affiliations:** Department of Emergency Medicine, Inselspital, University Hospital, University of Bern, 3010 Bern, Switzerland; mairi.ziaka@gmail.com

**Keywords:** acute respiratory distress syndrome, dysbiosis, fecal microbiota transplantation, gut–lung axis, gut microbiome, lung injury, lung microbiome

## Abstract

The gastrointestinal (GI) tract contributes significantly to the pathogenesis of acute respiratory distress syndrome (ARDS) by influencing systemic inflammation and sepsis, which are key factors in the development of multiple organ dysfunction syndrome (MODS), while the significant impact of gut microbiota in critically ill patients, including those with sepsis and ARDS, further underscores its importance. The intestinal microbiota is vital to immune system function, responsible for triggering around 80% of immune responses. Therefore, it may be hypothesized that modifying fecal microbiota, such as through fecal microbiota transplantation (FMT), could serve as a valuable therapeutic approach for managing inflammatory diseases like lung injury (LI)/ARDS. Indeed, emerging experimental research suggests that FMT may have beneficial effects in ARDS models by improving inflammation, oxidative stress, LI, and oxygenation. However, well-designed randomized clinical trials in patients with ARDS are still lacking. Our study seeks to examine how therapeutic interventions such as FMT might benefit LI/ARDS patients by exploring the interactions between the gut and lungs in this context.

## 1. Introduction

ARDS is marked by acute hypoxemic respiratory failure and bilateral infiltrates on chest imaging without the presence of cardiac failure or fluid overload. At the same time, one of its main pathophysiological features is inflammatory LI, leading to a global mortality rate near 40% [1]. In patients with ARDS, pulmonary vascular permeability and lung mass increase are observed in the presence of a reduction in aerated lung tissue [2]. Based on epidemiological evidence, it is estimated that over three million individuals are diagnosed with ARDS worldwide each year and that ARDS represents approximately 10% of intensive care unit (ICU) admissions [3].

The activation of the immune system, the formation of danger-associated molecular patterns (DAMPs), the activation of the innate immune response, the generation of neutrophil extracellular traps and histone release, the production of reactive oxygen species, leukocyte proteases, chemokines, and cytokines, and uncontrolled activation of coagulation pathways are typical dysregulated inflammatory mechanisms observed in ARDS [4,5,6,7]. In reference to the pathophysiology of ARDS, a major pathophysiological issue, apart from excessive inflammation, is the compromised lung microvascular barrier as a result of elevated endothelial and epithelial permeability [7,8,9].

Previous research indicates harmful organ crosstalk between the lungs and distant organs, ultimately resulting in MODS, which is the leading contributor to illness and death in patients with LI/ARDS [10,11]. Extensive research has highlighted the GI tract’s role in the pathogenesis of this syndrome [12], emphasizing its significant impact on critical conditions such as trauma, pancreatitis, hemorrhagic shock, burns, and ARDS. The GI tract influences systemic inflammation and the development of sepsis, crucially contributing to the pathophysiology of MODS [13,14,15,16,17]. Moreover, the substantial impact of gut microbiota in critically ill patients is widely acknowledged [13,14,18]. Indeed, prophylactic administration of antibiotics to suppress gut microbiota has been shown in studies to reduce the risk of multiorgan failure (MOF) and mortality in critically ill patients [19,20,21]. Experimental evidence strongly supports these findings, indicating that pre-treatment with antibiotics and using germ-free animals effectively reduces inflammation and injury in distal organs during shock [22,23,24,25,26].

Intestinal microbiota significantly contribute to the immune system’s function [27], initiating 80% of immune responses [11,28,29,30]. Regarding this matter, fecal microbiota modification, including FMT [31,32,33,34], may serve as a therapeutic tool for treating inflammatory diseases, including sepsis and LI/ARDS. Indeed, FMT has emerged as a method to restore the functions of disrupted gut microbiota by introducing fecal material from a healthy donor, gaining significant attention for its effectiveness in treating recurrent *Clostridioides difficile* (*C. difficile*) infection (CDI) compared to standard care [35]. Certainly, rescue FMT has become a promising alternative to surgical intervention for critically ill patients experiencing severe and complicated CDI, achieving a primary cure rate of nearly 80% and enabling nearly 90% of patients to avoid colectomy [36]. However, evidence supporting the efficacy of FMT in treating antibiotic-associated colitis (AAC) in critically ill patients caused by pathogens other than *C. difficile* or of unknown origin, which constitute approximately two-thirds of antibiotic-associated diarrhea (AAD) cases, remains limited [37,38,39,40]. Furthermore, recent research has demonstrated the beneficial effects of FMT in mitigating the progression of pulmonary inflammatory conditions like pulmonary fibrosis [41], suggesting a potential therapeutic role in managing other pulmonary inflammatory conditions, such as LI/ARDS. Yan and colleagues (2024) recently reported significant improvements in respiratory function in two late-onset classic amyotrophic lateral sclerosis (ALS) patients who required tracheostomy and mechanical ventilation (MV) following two rounds of FMT, resulting in successful weaning from MV [42]. This review aims to explore how gut–lung interactions influence ARDS pathophysiology and assess whether FMT could offer therapeutic potential in this setting.

## 2. Methods

We performed a comprehensive literature search using PubMed to identify relevant studies focusing on the gut–lung axis in LI/ARDS and on FMT as a therapeutic intervention, with particular emphasis on ARDS. The search terms included: “acute respiratory distress syndrome”, “lung injury”, “gut–lung axis”, “gut microbiome”, “intestinal microbiota”, “gut barrier disruption”, “inflammation”, “fecal microbiota transplantation”, “sepsis”, “critical illness”, “intensive care medicine”, and “critical care”. Boolean operators (AND, OR) and truncations were used to improve the search strategy. Articles published in English over the past 20 years (2005–2025) were included, while important seminal works outside this period were also incorporated to enhance foundational understanding. References from selected studies were manually screened to identify grey literature and additional eligible articles.

## 3. The Gut–Lung Axis in ARDS

### 3.1. Gut–Lung Microbiota Axis in ARDS

**Figure 1:**
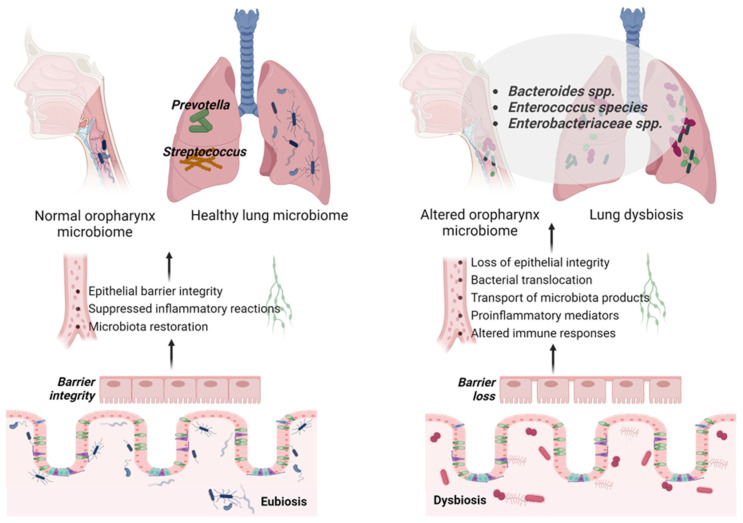
Impact of gut dysbiosis on gut–lung immunity and the lung microbiome. Figure created with BioRender.com.

The lungs and gut host the largest and most significant microbial communities in the human body, influencing health, disease severity, and mortality, while being linked through the gut–lung axis [18,43,44,45,46,47,48,49,50]. Indeed, in critically ill patients, major contributors to pathophysiological changes include intestinal dysbiosis, endotoxemia, and systemic inflammation, causing a depletion of beneficial microbiota and an overgrowth of potentially harmful bacteria. This, in turn, decreases short-chain fatty acid (SCFA) production or triggers an inflammatory response by the gut microbiota, ultimately resulting in prolonged immunosuppression and increased vulnerability to hospital-acquired infections [51,52,53]. Indeed, by maintaining normal immune balance and homeostasis, the commensal microorganisms in the lungs and gut are vital. When this balance is disrupted—a condition referred to as microbiota dysbiosis, characterized by changes in bacterial composition, abundance, and diversity—the host’s susceptibility to a wide range of infections can increase significantly (Figure 1).

**Figure 2:**
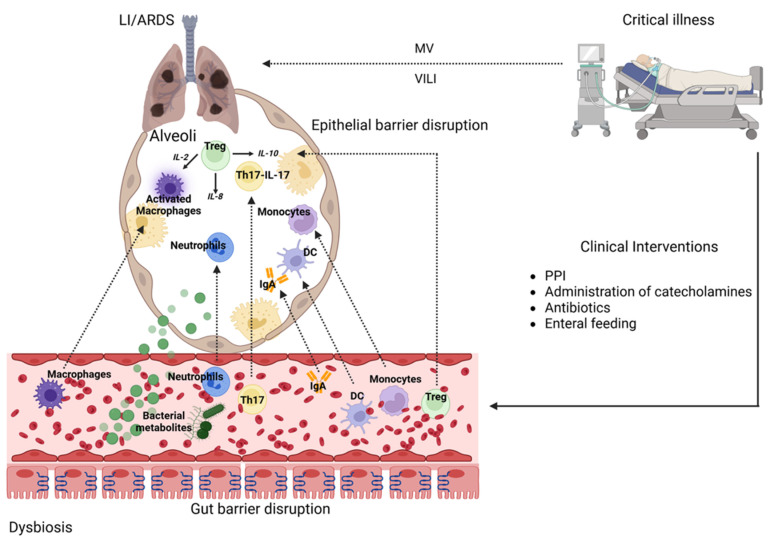
Simplified schematic representation of the gut–lung axis in critically ill patients. DC: dendritic cell; IgA: Immunglobulin A; IL: interleukin; PPI: proton pump inhibitor; Th17: T helper 17; Treg: regulatory T cell; VILI: ventilator induced lung injury. Figure created with BioRender.com.

Furthermore, such imbalances can exacerbate autoimmune diseases affecting the gut and lungs, enhance inflammatory responses, and contribute to a variety of metabolic disorders [54,55]. Additionally, in critically ill patients, multiple clinical interventions—such as enteral feeding, administration of proton-pump inhibitors, systemic catecholamines, and systemic antibiotic treatments—are commonly employed, each altering the environmental conditions for intestinal bacterial growth (Figure 2) [22]. As a result of the pathophysiological changes observed in ICU patients, microbial diversity becomes destabilized and frequently reduced, with critically ill patients sometimes having only four bacterial species identified [56,57]. The decrease in beneficial microbes, combined with the proliferation of potentially pathogenic and inflammatory bacteria, elevates the risk of hospital-acquired infections, sepsis, and MODS [28,58,59,60]. Furthermore, serving as a vital structural component of the gut, the mucus layer acts as a protective physical barrier. It supports its microbial community, thereby separating the intestinal environment from the host’s internal systems [22].

The intestinal barrier constitutes a major component of the anatomical and functional entity of the gut, and under normal conditions is responsible for defending the intestinal mucosa and preventing the translocation of bacterial components. It is composed of the intestinal microbiome, the mucosal immune system, and intestinal epithelial cells, which engage in complex crosstalk involving metabolic products, regulatory mediators, cytokines, and antimicrobial peptides, with the microbiome serving as the first line of defense [61,62,63]. The integrity of the intestinal mucosal barrier is altered in patients with ARDS, primarily due to gut microbiota dysbiosis, including reductions in beneficial bacterial populations such as *Bacteroides* and *Bifidobacterium* [64,65]. Moreover, in the context of sepsis-induced ARDS, cytokine cascades disrupt mucous layer integrity, resulting in hyperpermeability by impairing the expression of tight junction (TJ) proteins, altering the function of claudins, enhancing the activity of myosin light chain kinase, and regulating the proliferation and death of intestinal epithelial cells, leading to compromised paracellular transport and increased transmission of macromolecules [66,67,68,69]. In addition, during critical illness, reductions in protective metabolites, such as SCFAs, further contribute to intestinal barrier abnormalities and increased intestinal permeability [70]. Indeed, experimental research highlights the protective effects of SCFAs in lipopolysaccharide (LPS)-induced LI by modulating immune responses, suppressing inflammation, promoting the production of anti-inflammatory mediators, and regulating molecular pathways such as nuclear factor κB (NF-κB), thereby restoring intestinal integrity and improving LI [71,72,73,74]. In recent years, it has been proposed that in patients with ARDS and sepsis, impaired gut integrity leads to the translocation of gut microbes, such as *Enterobacteriaceae* and *Bacteroidetes*, across the intestinal barrier and into the lungs, facilitated by disrupted alveolo-capillary integrity, thereby further exacerbating LI (Figure 2) [18,75,76]. In addition, it has been shown that microbial metabolites can enter the systemic circulation, leading to the activation of immune and endothelial cells and contributing to the development or worsening of LI [11,77,78].

Patients with ARDS are frequently mechanically ventilated and therefore at risk of ventilator-induced lung injury (VILI), which can further enhance inflammatory responses and exacerbate intestinal injury and barrier disruption (Figure 2). Furthermore, hypercapnia resulting from lung-protective ventilation with low tidal volumes leads to increased myocardial contractility, reduced systemic vascular resistance, and impaired vascular tone, including splanchnic vascular tone, as well as compromised intestinal microcirculatory oxygenation, which may further impair barrier function [11].

A marked deterioration in gut barrier integrity during critical illness facilitates the translocation of intact microbes, microbiota products, immune cells, and pro-inflammatory mediators into the lungs, a process that further intensifies the systemic inflammatory response and contributes to the development of MOF [22,28,66,79]. Indeed, the production of inflammatory cytokines—including tumor necrosis factor (TNF)-α, transforming growth factor (TGF)-β, interleukin (IL)-5, IL-6, IL-1β, IL-13, IL-17, IL-18, and IL-33—and chemokines such as IL-8, chemokine ligand (CCL)2, CCL3, CCL4, CCL7, CCL20, CX-C motif chemokine (CXCL)5, CXCL8, and CXCL10 is triggered by increased systemic exposure to microbes and their metabolites from a dysbiotic gut [80]. As a result, pro-inflammatory immune cells, including neutrophils and T cells, are recruited to the mucosal epithelial layer, where they form lymphoid aggregates. Once activated, these cells migrate into the bloodstream and infiltrate various organs, such as the lung parenchyma (Figure 2) [28,81]. Emerging evidence highlights the significant contribution of the gut microbiota to maintaining immune homeostasis by modulating the critical balance between anti-inflammatory regulatory T cells (Tregs) and pro-inflammatory T helper 17 (Th17) cells, which play a central role in various inflammatory conditions affecting the lungs [28,82]. Indeed, accumulating research in recent years suggests that cellular components of the immune system, including Th17 and Treg cells of intestinal and bone marrow origin, contribute significantly to the pathophysiology of pulmonary inflammatory disorders, including ARDS, chronic obstructive pulmonary disease (COPD), asthma, sarcoidosis, and pulmonary infections [50,83,84]. In the context of an imbalanced gut microbiome, Treg activation, as well as the proliferation and differentiation of Tregs and Th17 cells, occurs, which triggers the intestinal production of IL-17 by Th17 cells and secretory immunoglobulin A (IgA) by B cells [85,86]. In addition, dysbiosis is associated with intestinal elevation of CD4^+^ IL-17-producing cells [87], whose immunoregulatory properties extend beyond the GI tract to affect distal organs and systems, including the lungs (Figure 2) [18,88,89].

Gut-derived pro-inflammatory factors can be transferred via the mesenteric lymphatics, potentially leading to systemic infection and inflammatory cascades and damage to distant organs, as the mesenteric lymph could act as a pathway for these harmful factors, which may contribute to the onset of ARDS [90,91,92]. This process is evidenced by the lung’s initial exposure to mesenteric lymph, where it encounters a high concentration of lymph components before being diluted by systemic blood. As intestinal lymph enters the systemic circulation through the thoracic duct, which then drains into the subclavian vein and subsequently the right heart, it is first encountered by the pulmonary vascular bed before entering the pulmonary circulation [91]. The disrupted interplay among the gut barrier, immune system, endogenous microorganisms, and the lungs, along with the associated increase in systemic inflammation, may potentially worsen or trigger LI/ARDS. To address this issue, gaining a more comprehensive understanding of the gut–lung axis could elucidate the complexities of these conditions and lay the groundwork for novel treatment strategies.

### 3.2. Gut Microbiome in ARDS

During critical illness, the normally diverse bacterial community in the lower GI tract can significantly decrease, resulting in the dominance of a few species, including *Pseudomonas aeruginosa* (*Ps. aeruginosa*), which is usually present in low quantities, as well as *Staphylococcus aureus* (*S. aureus*), *Enterococcus* spp., and members of the *Enterobacteriaceae* family (such as *Escherichia coli* (*E. coli*) and *Klebsiella* spp. (Figure 1) [22,56,57,58,93,94,95]. Indeed, recent studies convincingly demonstrated that within the first week in the ICU, critically ill patients exhibited a markedly different intestinal microbiota composition compared to healthy individuals, including an increase in *Enterobacteriales* and *Enterobacteriaceae*, which was associated with a 92% higher risk of adjusted mortality within 180 days [96]. A multi-center study found that patients with sepsis exhibited an elevated quantity of intestinal microbiota, with species such as *Parabacteroides*, *Fusobacterium*, and *Bilophila* significantly contributing to inflammation, while higher levels of *Enterococcus* spp. are linked to increased mortality [97]. Particularly in patients with ARDS, beneficial bacterial populations like *Faecalibacterium prausnitzii*, *Bacteroides*, and *Bifidobacterium*, which are vital for preserving the intestinal barrier and managing immune responses, are diminished. Concurrently, there is an increase in the prevalence of pathogenic or potentially pathogenic microorganisms, such as specific *Enterobacteriaceae* members, *C. difficile*, and various *Streptococcus* species [98]. Moreover, a recent study on the gut microbiome in patients with acute pancreatitis-related ARDS (AP-ARDS) revealed notable differences in microbiota composition and function compared to those with acute pancreatitis but without ARDS (AP-nonARDS). Specifically, the AP-ARDS cohort had elevated levels of the *Proteobacteria phylum*, *Enterobacteriaceae* family, *Escherichia-Shigella* genus, and *Klebsiella pneumoniae* (*K. pneumoniae*) while exhibiting a significant reduction in the *Bifidobacterium* genus. Furthermore, random forest modeling pinpointed the *Escherichia-Shigella* genus as a critical marker for distinguishing AP-ARDS from AP-nonARDS, suggesting its potential as a predictor for ARDS development in patients with acute pancreatitis. Additionally, a subgroup analysis revealed an association between intestinal microbiome composition at the early stages of acute pancreatitis and ARDS severity, indicating a possible pathogenetic role of preexisting microbiome alterations in both the development and severity of ARDS. Given that these alterations were observed at admission, they further suggest that preexisting intestinal imbalance may contribute to ARDS pathogenesis [99]. Moreover, in coronavirus disease (COVID)-19 patients, fecal microbiomes exhibited notable alterations, marked by an increase in opportunistic pathogens and a decrease in beneficial commensals, both during and after hospitalization. These microbiome disruptions persisted even after the virus was cleared and symptoms had resolved. Higher levels of *Coprobacillus*, *C. ramosum*, and *C. hathewayi* were linked to more severe disease, while *Faecalibacterium prausnitzii* was inversely associated with severity. Furthermore, *Bacteroides* species, which are known to reduce angiotensin-converting enzyme 2 (ACE2) expression, were inversely correlated with the severe acute respiratory syndrome coronavirus 2 (SARS-CoV-2) load in fecal samples during hospitalization [100]. Additionally, dysbiosis persisted in samples collected up to 30 days after disease resolution, as noted by the continued underrepresentation of gut commensals with known immunomodulatory functions, such as *Faecalibacterium prausnitzii*, *Eubacterium rectale*, and *Bifidobacteria*. This disrupted microbial composition was associated with varying degrees of disease severity and correlated with elevated levels of inflammatory cytokines and blood markers, including C-reactive protein, lactate dehydrogenase, aspartate aminotransferase, and gamma-glutamyl transferase [101]. Finally, dysbiosis in critically ill patients is marked by a decline in anaerobic bacteria, leading to lower levels of SCFAs, which play a cardinal role in maintaining the integrity of the gut barrier and promoting the host’s immune response. This decrease in SCFAs is associated with cellular apoptosis, malabsorption, diarrhea, and bacterial translocation [102,103,104,105]. Moreover, as above-mentioned, alterations in the microbiota can lead, among others, to immune system dysregulation, characterized by reduced IgA and T cell levels, while also affecting mucus secretion and the production of SCFAs that activate Tregs (Figure 1) [106,107,108,109]. Indeed, in a healthy physiological state, Th 1 and Th17 cells control the translocation of small amounts of bacterial products, such as polysaccharides from *Bacteroides* spp. and mucosa-adherent segmented filamentous bacteria. However, an excessive influx of bacteria triggers the overactivation of Toll-like receptors, leading to an overproduction of inflammatory cytokines, which damages the epithelial lining and results in chronic inflammation [110]. In the intestine, SCFAs are involved in several physiological processes, including maintaining homeostasis, enhancing epithelial barrier function, and facilitating the turnover of intestinal epithelial cells [111]. SCFAs stimulate mucin synthesis, preserve gut barrier integrity by inducing TJ assembly, exert immunomodulatory effects by promoting Treg cell differentiation, reduce local intestinal inflammation and permeability, and serve as a critical energy source for colonocytes, supporting their differentiation and proliferation [112,113].

### 3.3. Lung Microbiome in ARDS

Until recently, the widespread assumption of lung sterility [114,115] impeded systematic investigation of the lung microbiome, leading to delays in research advancements [116]. However, the discovery of complex and dynamic bacterial communities within the lungs has been made possible by recent advances in culture-independent microbiology [115]. The transition from eubiosis to dysbiosis in the lung may be associated with alterations in lung pathophysiology and impaired mucus clearance. At the same time, disruptions in the microbiome could further advance disease by amplifying inflammatory signals or disrupting cytokine production [11,117,118]. In critically ill patients, factors such as an impaired cough reflex, disrupted mucociliary clearance from endotracheal intubation and acute illness, and the presence of nutrient-rich alveolar edema—combined with pockets of oxygen and varying temperature gradients—create an environment that, along with host stress response signaling molecules, selectively fosters the growth of potential pathogens [22]. Moreover, compromised gut integrity facilitates bacterial translocation, whereby bacteria or their components breach the GI barrier and enter the bloodstream, resulting in the enrichment of the lung microbiome with gut-derived bacteria [18,119,120]. Indeed, experimental studies in sepsis models have highlighted that the lung microbiome becomes predominantly colonized by viable gut-associated bacteria. Moreover, utilizing culture-independent techniques to examine bronchoalveolar lavage fluid (BALF) from ARDS patients frequently reveals high levels of gut-specific bacteria, such as *Bacteroides* spp., *Enterococcus* species, and *Lachnospiraceae* species, which are not detected by conventional culture methods and are linked to increased systemic inflammation [18]. The same group confirmed that ARDS patients have a higher bacterial load, with BALF samples showing that while non-ARDS patients primarily have clusters such as *Streptococcaceae* and *Prevotellaceae*, ARDS patients have increased levels of *Pasteurellaceae* and *Enterobacteriaceae*. Notably, the presence of gut-derived *Enterobacteriaceae* was strongly linked to ARDS [121]. Furthermore, a markedly reduced α-diversity index in the pulmonary microbiome has been shown to correlate with both the duration of ICU stay and the need for MV, suggesting that alterations in the lung microbiome may contribute to the pathogenesis of septic LI/ARDS [122]. Finally, significant changes in lung microbiota composition occur shortly after ICU admission in critically ill patients and are associated with the development of ARDS [75].

On the other hand, lung dysbiosis may significantly influence the composition of gut microbiota [11]. A study analyzing samples from 40 patients infected with the H9N2 avian virus revealed decreased microbial diversity, accompanied by an overgrowth of *E. coli* and *Enterococcus faecium* (*E. faecium*). Furthermore, *Eubacterium*, *Ruminococcus*, *Bifidobacterium*, and *Roseburia* levels were significantly reduced in these patients [123]. In a preclinical study, influenza infection was shown to increase the presence of *Enterobacteriaceae* while decreasing the levels of *Lactobacilli* and *Lactococci* in the gut [46]. A cross-sectional study using 16S ribosomal RNA (rRNA) techniques to examine the intestinal microbiota in 30 COVID-19 patients and 24 influenza A (H1N1) patients revealed that those with COVID-19 had notably reduced bacterial diversity and an increased relative abundance of opportunistic pathogens, including *Streptococcus*, *Rothia*, *Veillonella*, and *Actinomyces*. Additionally, beneficial symbionts were less prevalent in these patients. In contrast, H1N1 patients had lower levels of *Actinobacteria*, *Erysipelotrichia*, *Clostridia*, and beneficial butyrate-producing families such as *Lachnospiraceae* and *Ruminococcaceae* [124].

The emerging understanding of lung microbiome dynamics highlights its complex interaction with gut microbiota and its role in critical illness. The shift from a previously assumed sterile environment to a more intricate microbial ecosystem underscores the need for further research into how lung dysbiosis and associated microbial imbalances contribute to disease processes such as ARDS.

## 4. Therapeutic Implications and Area of Further Research

### Fecal Microbiota Transplantation

Accumulating evidence in recent years indicates that modulating dysbiosis through microbial therapies, including probiotics and FMT, may help prevent and treat respiratory diseases via multiple mechanisms, such as reducing intestinal inflammation, enhancing mitochondrial function, and suppressing lung inflammation, among others [125,126,127]. Apart from the potential application of FMT in non-small cell lung cancer, COPD, and asthma [128,129,130], studies report the use of FMT in patients with severe pneumonia by modulating harmful bacterial populations, normalizing metabolic pathways, and restoring the intestinal microbiome [131,132]. The existing data in patients with ARDS are sparse; however, given that immune dysregulation contributes significantly to its pathogenesis and that the gut microbiota influences immune responses in ARDS, FMT is hypothesized to have therapeutic potential in this condition [133].

FMT refers to the procedure where gut bacteria from a healthy donor are introduced into a patient to re-establish a stable microbial community in the gut. By swiftly changing the intestinal microbiota composition, FMT has demonstrated its effectiveness and shows potential as a treatment for numerous health conditions linked to gut dysbiosis [134,135]. Interest in FMT experienced a marked surge in 2013, coinciding with the publication of a randomized controlled trial demonstrating its superior efficacy to conventional therapies for recurrent CDI. Historically, FMT has been acknowledged for its therapeutic benefits in treating conditions such as food poisoning and dysentery. However, the findings of a randomized controlled trial presenting FMT’s substantial superiority over standard care by treating severe *C. difficile* colitis, where it showed a success rate of nearly 90%, significantly renewed attention and provided robust validation for the potential application of FMT in contemporary medical practice [28,35,136,137,138]. Moreover, FMT has been demonstrated as a successful treatment option for other GI diseases, such as inflammatory bowel disease [139]. Additionally, it shows promise for treating non-GI diseases, including neurological and psychiatric disorders, among others, acute ischemic stroke [87,140], autism [141,142], and Parkinson’s disease [143]. Since the microbiome is disrupted during critical illness, leading to MODS, FMT is being investigated as an additional therapy in the management of critical illness, including sepsis. The initial documented case involved a critically septic patient experiencing severe dysbiosis who suffered from persistent sepsis and watery diarrhea for 30 days post-vagotomy despite receiving antibiotics and supportive therapy. Administration of donor FMT resulted in the resolution of both sepsis and diarrhea. Subsequent molecular analyses verified the reconstitution of the patient’s microbiome with healthy bacteria (Table 1) [38]. Moreover, Wei and co-workers (2016) presented two patients with hemorrhagic and ischemic stroke who developed MODS, septic shock, and severe diarrhea. After confirming intestinal dysbiosis, the patients received FMT, which led to the resolution of their symptoms. Microbiota analysis post-treatment showed increased *Firmicutes* and decreased *Proteobacteria*, correlating with normalized stool output and reduced inflammation markers [39]. In 2017, Wurm et al. documented a case of a critically ill patient presenting with severe antibiotic-associated apoptotic enterocolitis. Microbiota analysis revealed profound depletion of gut microbiota accompanied by overgrowth of opportunistic pathogens. The patient underwent FMT, which was associated with the restoration of microbial balance (correction of dysbiosis), rapid clinical improvement, and resolution of enterocolitis (Table 1) [37]. A case series study focused on the adverse events, safety, and outcomes for critically ill patients with AAD found that 38.9% (7 out of 18) experienced adverse events related to FMT. These events included abdominal pain, increased diarrhea frequency, amylasemia, and fever during a follow-up period of at least 12 weeks. There were no reported deaths or infectious complications attributed to FMT. Additionally, 72.2% (13 out of 18) of the patients experienced an improvement in abdominal symptoms within one week, and 44.4% (8 out of 18) achieved successful outcomes following rescue FMT [40].

The data on the use of FMT in patients with LI/ARDS are limited. A recent comparative, retrospective, single-center study involving 86 patients with concurrent COVID-19 and severe pseudomembranous colitis highlighted that patients who received a combination of FMT and antibiotics as rescue therapy for colitis exhibited a notable reduction in inflammatory markers (C-reactive protein and white blood cell count) compared to those who received only antibiotics. Additionally, these patients experienced a lower relapse rate and a reduction in abdominal symptoms (91.3%). Conversely, co-infected patients who were treated solely with antibiotics exhibited higher levels of fibrinogen, persistent moderate abdominal pain (82.5%), and a significantly higher relapse rate of CDI (42.5%) [144]. Furthermore, Yan and colleagues reported on two patients with ALS and severe respiratory failure who were mechanically ventilated and received two rounds of FMT as rescue therapy for their refractory ALS and respiratory distress. Analysis of fecal samples from both patients before and after FMT revealed changes in gut microbiota diversity, with *Firmicutes* and *Actinobacteria* being the predominant phyla. Specific bacterial taxa included *E. faecalis*, *Collinsella aerofaciens*, *Bacteroides stercoris*, *K. pneumoniae*, and *Bacteroides uniformis* in the first patient, and *E. faecium*, *E. faecalis*, *C. clostridioforme*, *C. innocuum*, *C. michiganensis*, and *Flavonifractor plautii* in the second patient. Increased α-diversity was observed after the second FMT, reducing the disparity between the patients’ “pathobiome” and the donor microbiome. The authors reported significant improvements in respiratory mechanics following the two FMT procedures, including the reduction in ventilator support from invasive to noninvasive modes and an increase in the total ALS Functional Rating Scale-Revised (ALSFRS-R) score. They concluded that FMT could have therapeutic potential for managing advanced ALS and respiratory failure (Table 1) [42]; however, the current evidence is limited, and further well-designed randomized controlled trials are required to evaluate its efficacy and safety.

Experimental studies further validate these findings (Table 1). A recent study examined how gut microbiota influences pulmonary fibrosis in C57BL/6 mice, focusing on comparisons between substrains (C57BL/6J and C57BL/6NCrl) sourced from different suppliers. The study employed germ-free models, FMT, and cohousing techniques to facilitate gut microbiota transfer between the mice. The researchers identified distinct keystone species specific to each substrain through metagenomic analysis. The findings indicated that gut microbiota significantly impacted the development of pulmonary fibrosis. Notably, C57BL/6NCrl mice exhibited lower levels of lung fibrosis and reduced mortality rates, as well as an increase in CD4+ IL-10 T cells and a decrease in IL-6 levels. Furthermore, the horizontal transmission of microbiota via cohousing led to decreased mortality in C57BL/6J mice and induced changes in their pulmonary immune response. [41]. These findings are supported by additional experimental research demonstrating that FMT from normal rats mitigates LPS-induced LI by modulating the gut microbiome, suppressing inflammatory mediators such as TNF-α, IL-1β, and IL-6, reducing alveolar epithelial damage and increasing partial pressure of arterial oxygen (PaO_2_) [145]. These results are consistent with those of Tang et al. (2021), demonstrating that FMT reduced oxidative stress and inflammation in an experimental dysbiotic model of LPS-induced LI by modulating the Toll-like receptor 4/nuclear factor kappa B (TLR4/NF-κB) signaling pathway [146]. Similarly, in a mouse model of LI caused by Influenza A infection, FMT mitigated virus-induced inflammatory LI [147]. In an experimental model of LPS-induced LI in rats, Yin et al. (2019) found that FMT reduced LI and suggested that this impact could be linked to the inhibition of PI3K/AKT/NF-κB signaling pathway activation and the decreased expression of the inflammatory factor intercellular adhesion molecule 1 (ICAM-1) [148]. Moreover, it has been shown that lung–gut microbiota modulation through human umbilical cord mesenchymal stromal cells (HUC-MSCs) can alleviate LPS-induced LI, and that FMT from HUC-MSC-treated animals limits LI in LPS-mediated damage, also by modulating the TLR4/NF-κB signaling pathway [149]. Recent research in a rat model of LPS-induced ARDS demonstrated that FMT modulates the expression of retinoic acid receptor-related orphan receptor gamma t (RORγt) and Forkhead box P3 (Foxp3), reestablishing the Th17/Treg cell ratio through inhibition of the Janus kinase/signal transducer and activator of transcription (JAK/STAT) signaling pathway, which in turn resulted in improvements in both intestinal and lung injury [133].

**Table 1:** Evidence for the therapeutic potential of FMT in animal models of LI/ARDS and in critically ill patients, including ARDS, non-CDI sepsis, and severe non-CDI infections. ALS: amyotrophic lateral sclerosis; ARDS: acute respiratory distress syndrome; CDI: *Clostridioides difficile* infection; FMT: fecal microbiota transplantation; LI: lung injury; MODS: multiple organ dysfunction syndrome; pts: patients; RCT: randomized controlled trial.

Evidence Source	Model/Study Type	Key Findings	References
Preclinical studies	Animal models of LI, pneumonia, and ARDS	FMT restored gut–lung immune balance, reduced inflammation and fibrosis, improved oxygenation, and decreased mortality.	[41,133,145,146,147,148,149,150,151]
Human studies in LI/ARDS	Case report	Severe ARDS with refractory CDI; FMT via upper endoscopy resolved diarrhea. Data on ARDS outcomes were not reported.	[33]
Human studies in severe pneumonia	Case report	95-year-old with severe pneumonia and pan-drug-resistant *K. pneumoniae*; FMT led to respiratory improvement and clinical recovery after treatment failure.	[132]
Human studies in respiratory failure, other etiology (ALS)	Case series, RCT	RCT (27 pts): No significant effect of FMT on respiratory function. Case series: Marked respiratory improvement and ventilator weaning after FMT.	[42,152]
Human studies in sepsis, MODS, and severe infection	Case reports, case series	FMT restored gut microbiota, alleviated systemic inflammation, and improved clinical status; respiratory outcomes were not always explicitly reported, but overall organ function and recovery were supported.	[37,38,39,40,153,154,155,156,157,158]

Furthermore, FMT in an ARDS rat model leads to significant alterations in the gut microbiome, particularly affecting the composition of *Lactobacillus* and *Akkermansia*, while restoring *Clostridia_UCG-014*, *Muribaculaceae*, *Adlercreutzia*, and *Prevotella*, and decreasing *Romboutsia.* Moreover, FMT has been shown to regulate lipid metabolism, amino acid biosynthesis, and immune pathways, resulting in improved LI and respiratory function, increased PaO_2_, and decreased partial pressure of arterial carbon dioxide (PaCO_2_) [150]. Interestingly, in a mouse model of *Ps. aeruginosa* pneumonia, Wen et al. (2022) reported that FMT alleviated gut microbiota dysbiosis, corrected the Treg/Th17 cell ratio, mitigated inflammatory reactions and tissue injury, and inhibited the LPS biosynthesis pathway [151]. This research underscores the essential role of gut microbiota in modulating immune responses and maintaining lung health, highlighting particular microbial communities as key defenders against LI and fibrosis and suggesting new avenues for potential therapeutic approaches in ARDS treatment.

Despite the promising potential of FMT in the management of severe *C. difficile* colitis and other conditions, including severe LI and ARDS, there remain major concerns about potential side effects, which may be underestimated [159]. A systematic review and meta-analysis conducted by Marcella et al. (2021) [159], which included 129 studies and 4241 patients who underwent FMT, reported FMT-related side effects in 19% of patients. Diarrhea and abdominal discomfort or cramping were observed in 10% and 7% of cases, respectively. Serious adverse events occurred in 1.4% of patients, including death and infectious complications, with the authors noting that all severe events occurred in patients with mucosal barrier injury [159]. Importantly, another meta-analysis comparing adverse events of FMT in immunocompromised patients reported side effects similar to those observed in immunocompetent patients [160]. Nevertheless, the long-term consequences of FMT remain a concern, and the available evidence is currently limited, predominantly focusing on bacterial species, even though other intestinal entities, such as viruses, are more prevalent [161,162,163]. Moreover, several studies have thoroughly investigated the longevity of the transplanted microbiome and its immune interplay with the host, as not all bacterial identities are beneficial to the recipient [164]. A further concern is that the intestinal microbiome in patients who receive FMT continues to change over long periods of time [165], with unpredictable consequences for the host [164], such as the potential development of chronic and/or autoimmune diseases associated with specific microbiome profiles [166,167]. In addition, the transfer of antibiotic resistance genes and virulence factors should be carefully considered, as antibiotic resistance can be transmitted among intestinal bacterial species [168,169,170]. Indeed, although it is considered a rare complication, bacteremia with multidrug-resistant bacteria has been reported after FMT and can also be fatal [171].

The interpretation of existing evidence should be approached with caution, as most studies have small sample sizes, which can lead to random error and conflicting outcomes. In addition, there is substantial heterogeneity across studies regarding therapeutic interventions, including antibiotic therapy and MV, as well as in the definitions of pulmonary inflammatory response, microbiome alterations, and diversity [172]. Methodological heterogeneity and incomplete reporting of FMT protocols also extend to donor characteristics—such as age and sex—as well as to preparation procedures, including variations in sample processing, storage conditions, dosage, administration route, and delivery technique [173,174]. Furthermore, clinical outcomes differ between studies, making it difficult to draw conclusions about the relationship between microbiome alterations and prognosis. Unfortunately, as noted in a systematic review and meta-analysis investigating the composition and diversity of the pulmonary microbiome in ARDS, many of the existing studies are of poor quality and involve heterogeneous ARDS populations [172]. In addition, as the vast majority of studies report positive effects of FMT, there is a concern about potential publication bias regarding its safety and efficacy, since negative or statistically non-significant results may remain unpublished [174,175]. Although the overall safety profile of FMT appears generally favorable [159], another concern in critically ill patients is the risk of procedure-related complications. These patients are particularly vulnerable because the integrity of the intestinal mucosal barrier is often compromised, gut immunity is impaired, and vasoconstriction-related ischemic changes may promote bacterial translocation, thereby raising important safety considerations [175,176]. Beyond clinical risks, FMT in ARDS patients raises ethical concerns, as this highly vulnerable population, often sedated, mechanically ventilated, and neurologically impaired, is frequently unable to make autonomous decisions, understand safety information, or provide informed consent, particularly given the off-label use of the procedure [177,178]. As the existing evidence regarding FMT in critical illness, mainly derived from case reports, small series, or animal models, remains experimental and cannot be directly extrapolated to humans, future research must address the complex challenge of identifying specific mechanisms within the intricate microbial ecosystem involved in FMT. To enhance understanding and improve treatment outcomes, more targeted trials focusing on the precise pathophysiologic mechanisms of FMT are essential. These studies should aim to elucidate the different microbial communities’ specific roles and interactions, ultimately refining FMT protocols and expanding their therapeutic potential. Given the poor quality and heterogeneity of most existing studies, as well as the predominant use of open-label designs, further double-blinded, placebo-controlled studies with larger populations are needed to accurately assess the clinical efficacy of FMT in *C. difficile* colitis [179] and other conditions, along with its potential complications and side effects. Finally, in the authors’ opinion, given the well-established knowledge of ARDS subphenotypes [180,181], exploring distinct ARDS patient populations with specific gut microbiome alterations—where FMT could offer therapeutic benefit—may open new research and treatment horizons in the management of ARDS. In summary, the current evidence base surrounding FMT in ARDS remains highly preliminary and should be interpreted with considerable caution. The combination of small sample sizes, substantial methodological and protocol heterogeneity, limited ARDS-specific data, and the likelihood of publication bias significantly restricts the reliability and generalizability of existing findings. Safety and ethical concerns further under-score the need for rigorous evaluation before FMT can be considered for broader clinical application in this population. Overall, these limitations highlight a critical need for well-designed, adequately powered, and protocol-standardized clinical studies to more definitively assess the role of FMT in ARDS.

## 5. Conclusions

In conclusion, investigating FMT as a potential treatment for ARDS is promising, but it remains highly experimental. While FMT is well-established for treating gut dysbiosis and recurrent CDI, its application in critical illnesses like LI/ARDS is still under-researched. Initial evidence indicates that FMT may benefit lung health by modulating systemic inflammation and restoring microbial balance. However, more focused experimental research is necessary to understand its mechanisms and refine treatment approaches. At present, FMT is not recommended for ARDS outside of clinical trials, and its use should be approached with caution. Given the supportive nature of current ARDS management and the persistently high mortality rates, FMT may represent a novel adjunctive therapy, pending results from controlled trials. Well-designed experimental studies and randomized controlled trials are needed to further clarify its therapeutic potential in LI/ARDS.

## Data Availability

Not applicable.
